# PARS: Using Augmented 360-Degree Panoramas of Reality for Construction Safety Training

**DOI:** 10.3390/ijerph15112452

**Published:** 2018-11-03

**Authors:** Ricardo Eiris, Masoud Gheisari, Behzad Esmaeili

**Affiliations:** 1 Rinker School of Construction Management, University of Florida, Gainesville, FL 32611, USA; reiris@ufl.edu; 2 Sid and Reva Dewberry Department of Civil, Environmental, and Infrastructure Engineering, George Mason University, Fairfax, VA 22030, USA; besmaeil@gmu.edu

**Keywords:** 360-degree panoramas, augmented panoramas of reality, hazard recognition, construction safety training, virtual reality

## Abstract

Improving the hazard-identification skills of construction workers is a vital step towards preventing accidents in the increasingly complex working conditions of construction jobsites. Training the construction workforce to recognize hazards therefore plays a central role in preparing workers to actively understand safety-related risks and make assertive safety decisions. Considering the inadequacies of traditional safety-training methods (e.g., passive lectures, videos, demonstrations), researchers have employed advanced visualization techniques such as virtual reality technologies to enable users to actively improve their hazard-identification skills in a safe and controlled environment. However, current virtual reality techniques sacrifice realism and demand high computational costs to reproduce real environments. Augmented 360-degree panoramas of reality offers an innovative alternative that creates low-cost, simple-to-capture, true-to-reality representations of the actual construction jobsite within which trainees may practice identifying hazards. This proof-of-concept study developed and evaluated a platform using augmented 360-degree panoramas of reality (PARS) for safety-training applications to enhance trainees’ hazard-identification skills for four types of sample hazards. Thirty subjects participated in a usability test that evaluated the PARS training platform and its augmented 360-degree images captured from real construction jobsites. The usability reviews demonstrate that the trainees found the platform and augmentations advantageously to learning hazard identification. The results of this study will foreseeably help researchers in developing engaging training platforms to improve the hazard-identification skills of workers.

## 1. Introduction

The backbone of any occupational health and safety discussion is hazard identification; however, current lecture-based and passive methods of teaching hazard identification are losing their relevancy. The emergence of a new technology-savvy generation obliges instructors to abandon passive means of teaching and allocate more emphasis on creating engaging learning experiences that adopt virtual technologies and digital sites [1]. Virtual reality (VR) technology can create active learning experiences that engage the learners, increase learning retention scale comparing to passive learning practices, and most effectively provide them with on-demand learning opportunities for deliberate practice [2]. Virtual jobsites have been presented as a promising tool for safety education, but the effectiveness of harnessing these digital construction sites have not yet been fully investigated for educational purposes. In particular, a gap in knowledge remains about the use of real construction projects—as compared to computer-generated virtual representations of the environments—to create true-to-life training experiences for construction workers. Currently, virtual modeling methods such as Building Information Modeling (BIM) provide media to visualize components and to manage and coordinate associated construction-management activities. These virtual models simulate and address the replacement of full, real-world conditions in terms of time, physical space, and material properties. However, these models hinder and diminish the full perception of the real-world environment, providing users with an unrealistic, computer-generated simulation of a construction environment that does not render all the dynamic elements that may be at play on a real-world jobsite.

One emerging technology that can address this limitation are augmented panoramas of reality (PARS) for construction-safety training. Unlike common virtual environments that provide computer-generated simulations that do not replicate the details of real environment, augmented panoramas of reality create highly realistic and detailed representations of actual construction sites while giving users a sense of immersion. These features enable augmented panoramas of reality to become a strong tool for developing training materials specifically for construction safety. In such an environment, construction workers and professionals are capable of navigating within the data-rich environment of a real construction project to observe and identify the safety challenges in various spots.

Accordingly, this proof-of-concept research project first developed augmented 360-degree reality panoramas of reality platform that allows superimposition of information layers over complex situations in construction sites. Subsequently, the 360-degree panoramic images from real construction jobsites were populated with safety-related layers of information based on the OSHA and Susan Harwood materials for hazard identification. Ultimately, this study conducted a usability test with 30 participants to determine whether this platform can provide an easy-of-use method for learning safety-related concepts and to gain insides on the benefits of the platform as a hazard identification and training experience.

## 2. Background

### 2.1. Construction Safety Training and Hazard Identification

Considering that potential hazards on construction jobsites increase the chances of an incident [3,4], the ability to identify hazardous conditions before initiating a working task is an indispensable tool to achieve proactive safety management and risk mitigation. However, since it is almost impossible to eliminate all hazards from a working environment, construction workers themselves must be able to identify hazards and make proper decisions to avoid accidents. As a result, awareness and identification of hazards is the basis of any robust construction safety program [5,6].

Training programs are often used by employers to improve the hazard-recognition skills of their personnel [7]. Insufficient safety training has been consistently identified as a leading attribute in accident occurrence [7,8,9]. In particular, hazard-recognition training is of critical importance because it raises workers’ awareness of common risks on the construction jobsite by transferring safety-related knowledge to the workforce. Ultimately, this training encourages workers and professionals to make safety-conscious decisions, minimize risk, and avoid potential injuries. Consequently, scholars and industry professionals agree that safety training is paramount to enhancing hazard recognition on complex and dynamic jobsites [7,9].

While important research efforts have been undertaken in the past to advance this type of intervention e.g., [10,11], studies demonstrate that deficiencies remain within the commonly applied training methods. First, some of the issues attributed to inefficiencies of safety training have been linked to the inherent characteristics of construction as an industry: The short-term nature of construction employment, companies’ variable safety cultures and training budgets, and the difficulties involved in demonstrating the benefits of safety all have significant impacts on the effectiveness of training programs [12,13,14,15,16]. Second, deficiencies specific to the design of traditional safety training methods have been identified in previous studies. Low levels of engagement in safety training (lectures, videos, or demonstrations) have been proven to provide minimal efficiency in conveying safety-related knowledge, including hazard-identification skills [11,17,18]. Third, current safety-assessment techniques can contribute to poor safety performance in construction projects, a reality resulting from the challenge of translating an assessment’s static in-text descriptions of complex safety problems (i.e., construction process, location and site environment) to knowledge about dynamic real-life situations [19]. Combined, these factors indicate that a large percentage of hazards remain unrecognized in the workplace [20,21].

Additionally, a recent study showed that traditional training programs—even those generally accepted within the industry—still suffer limitations in helping workers acquire hazard-recognition skills. Hasanzadeh et al. [22] used eye-tracking technology to measure the impact of safety knowledge (in terms of training, work experience, and injury exposure) on construction workers’ attentional allocation toward jobsite hazards. Their study found that although work experience and injury exposure significantly impact visual search strategies and attentional allocation toward hazards, the difference between workers with and without the OSHA 10-h certificate was not significant. While the results did not state that the OSHA 10-h certificate is ineffective, the study revealed the need for developing more innovative training techniques, such as the high-engagement methods for safety training (e.g., trainee-centric, highly interactive) that have been proposed in recent years [9,23]. Hasanzadeh et al.’s [22] results underscore the potential benefit of integrating both tacit knowledge (work experience and injury exposure) and explicit knowledge (e.g., interactive training) to enhance worker safety.

### 2.2. Application of Virtual Reality in Construction Safety Research

In response to the shortcomings of traditional safety training, academia has explored the use of virtual reality (VR) to create active learning experiences that engage the learner. VR has been used to provide training opportunities for dangerous tasks in the jobsite, allowing users to avoiding exposure to potential harm. Researchers in the domain of safety have designed VR systems as serious games to achieve effective learning within digital environments, promoting user motivation and active engagement in their instructional practices as summarized in Table 1.

Although these VR simulations mediate to replace real-world conditions—time, physical space, and material properties, these environments are uncapable of presenting a full experience of real working conditions. VR has limited capabilities of delivering high degrees of realism on which trainees might not perform with the same proficiency in real world operations as they do in the simulated realm [30]. Additionally, VR requires large amounts of resources from the development perspective and from the end-user perspective. Modelling close-to-reality settings necessitates significant efforts in terms of time to achieve a sufficiently realistic representation of reality and often entails high computational cost for the rendering of all the elements on each scene [9,19,26]. Consequently, VR suffers from a low agility in the face of evolving work environments.

### 2.3. Why Use 360-Degree Panoramas?

360-degree panoramas create an unmodeled view of real environments that looks identical to reality, which provides inherent benefits over traditional virtual reality techniques. VR’s complex, real-world simulations are very computationally intensive and time consuming, since computer-generated representations of the environment are modeled from a user’s perception of reality. Furthermore, while VR’s 3D computer graphics allow users to synthesize an environment for arbitrary representations, the rendering quality and scene complexity are often limited because of real-time constraints [31]. Alternatively, the capturing technologies for building 360-degree panoramas provide unbroken views of a whole region surrounding an observer, giving a “sense of presence, of being there” [32] to the observer. Thus, 360-degree panoramas offer low computational-cost, easy-to-capture, non-computer-generated simulations that are beneficially immersive to the user due to the realism embedded in the photography and videography data.

Interactive panoramic scenes have also been used for several applications by researchers in the construction domain. Early research focused on addressing the technicalities of creating, capturing, interpreting, and navigating 360-degree images and video: Finch and Wing [33] employed video-still images on a computer-based system to produce a navigable simulator for students in built environment disciplines. Mei and Wing [34] used a series of interconnected 360-degree panoramic images that enabled users to navigate from one image to another through an interface to visit virtual construction sites. Dickinson et al. [35] developed a computer-based learning resource that used overlapping images to explore a virtual panoramic site.

Several research projects have used 360-degree scenes as a reality backdrop upon which to augment mainly 3D models [36,37,38,39]. In one such example, Côté and his colleagues [36] used a panorama of the surface of a street to augment a virtual excavation and illustrated underground utilities over the images. Other forms have used augmented panoramas in architectural and real estate domains to create virtual walk-throughs of real environments for clients; in these applications, panoramic images or videos were taken of an interior and were then augmented with various types of information (e.g., virtual 2D signs, audio, or 3D models) to create a natural tour of the building for potential buyers. Additionally, 360-degree panoramas have been used as method to provide construction managers with a method to record the building process in the jobsite. Eiris et al. [40] described the process of using modern capturing methods for 360-degree panoramas to create a virtual tours of complex construction projects for asset management and documentation.

More recently, 360-degree panoramas have been used as safety-training tool to enables visualization of hazards. Table 2 illustrates each of these applications used to deliver an immersive experience of interacting with a real space in a virtual environment using this technology in the construction-safety area.

## 3. Motivation and Point of Departure

As discussed above, previous research has found that construction-safety training yields low levels of engagement, which diminishes the benefit of these interventions. Alternatively, scholars have presented VR as a method to increase safety-related knowledge retention. Nevertheless, using VR to represent complex simulations of the real-world is currently very computationally intensive and time consuming. Although 3D computer graphics allow users to synthesize an environment for arbitrary representations, realism is often constrained by the rendering quality and scene complexity.

Although the contribution of studies found in the literature in the creation of training materials and contents is outstanding, none of the methods found provide a realistic connection between the virtual training and work environment of the construction jobsite. This limitation stems from the fact that replicating the complex conditions of an as-built work environments in virtual reality setting is computationally expensive and time-consuming [1]. To address this limitation, 360-degree panoramas of reality have been used in recent research studies to provide immersive representations of construction jobsites for safety-training purposes. This proof-of-concept study builds on the outcomes of previous studies [42,43] utilizing 360-degree panoramas of reality with layers of augmented information and defined a graphical user interface that is conducive for improving workers’ hazard-identification skills within the complex context of real construction projects. Within the platform, trainees actively practice identifying hazards in a highly engaging and realistic environment. These low-cost, simple-to-capture representations of real settings provide unbroken views of a whole region surrounding an observer, thereby allowing for an interactive look-around experience with a strong sense of presence. The contribution of this research is to present an alternative method for practicing hazard identification using 360-degree panoramas of the actual construction jobsite, thereby enabling training opportunities that better train users to recognize four sample types of hazards.

## 4. The PARS Platform

Before discussing the materials and methods, the research team undertook the creation of the user-experience within this proof-of-concept study; the authors have included a general description of the considerations underpinning the PARS platform. Specifically, the platform developed in this study employs 360-degree panoramic images that have been augmented with safety data for the trainee to engage in active exploration. Trainees practice identifying hazards over three sessions—Training, Assessment, and Feedback, with the objective of learning, testing, and receive comments about their acquired knowledge.

### 4.1. Platform Architecture and Data Management

**Figure 1:**
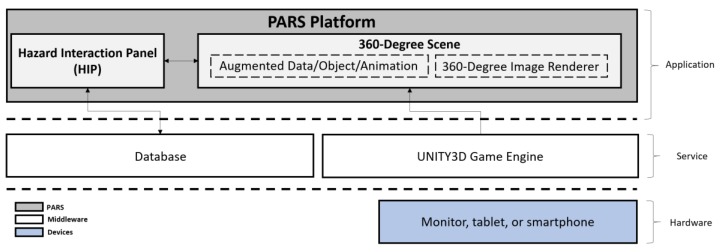
Platform Architecture—Application, Service, and Hardware Layers.

The safety training platform contains three distinct layers: application, service, and hardware (Figure 1). The layers represent the basic elements required by the platform to function.

The trainee only has access to the application layer, where all the training interactions occur. In this layer, the trainee observes and identifies hazards, and then receives feedback about the hazard-recognition tasks. The application layer includes two functional blocks: the hazard identification panel (HIP) and the 360-degree scenes. The HIP serves as the interactive space within which the user engages the platform. The 360-degree scenes include renderings of 360-degree panoramic images and layers of safety information in the form of augmentations. These augmentations include annotations in the form of data, objects, animations, or sounds.

The service layer consists of the digital tools employed by the application layer to enable and support the platform’s activities. Specifically, the platform utilizes the Unity3D^®^ (Unity Technologies, San Francisco, CA, USA) game engine, and a database that contains trainee information for each session (e.g., time spent reading information, interactions with interface, hazard selections, etc.). Unity3D^®^ is the middleware upon which this study developed the platform. The service layer employs the JavaScript Object Notation (JSON) data structure to build the database of trainee information, as it easy to interpret, requires minimal setup, and can be accessed in any type of device. The locally stored data in the database captures the trainee’s interactions and selections on the training and assessment sessions, and enables instantaneous comments by automatically processing the collected data in the feedback session. Additionally, this data permits the research team to posteriorly analyze the trainee’s interactions and selections while using the platform, gaining insides on hazard identification activities and platform usage patterns. The last layer contains the hardware devices that allow the trainees to physically engage the platform. Currently the platform supports the visualization with a monitor, tablet, or smartphone, and the platform functions as a standalone, locally executed software.

**Figure 2:**
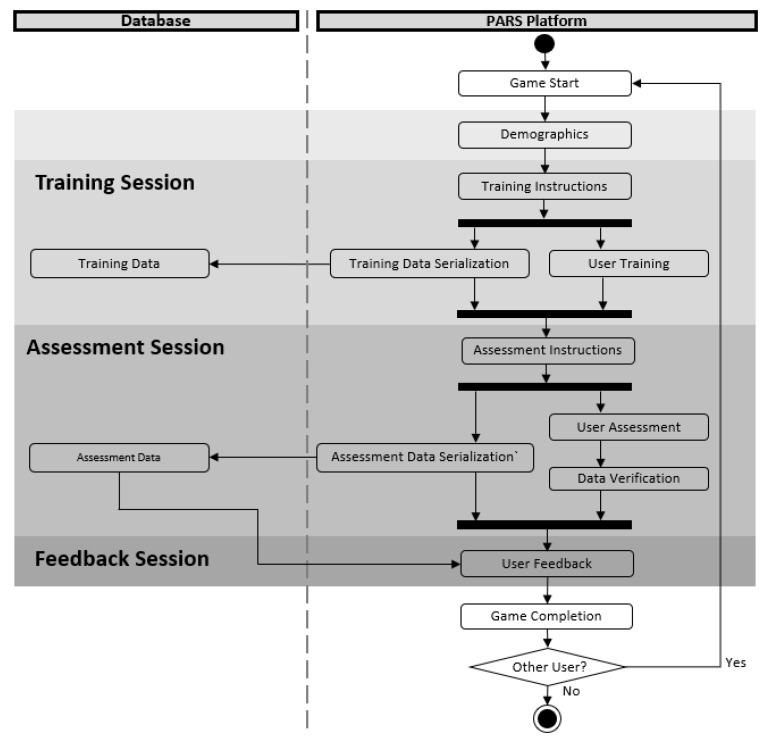
Platform Data Management using an UML diagram.

As illustrated in Figure 2, the data management of the platform proceeds according to an activity-based unified modeling language (UML) diagram that runs within the Unity3D application and get supported by the database.

In order to allow the user to learn and receive information about construction safety hazards, each successive interaction with the game triggers data transfer from the Training Session, Assessment Session, or Feedback Session into the database and vice versa. Each of these sections drives the tasks required from the trainee at different stages of the software utilization. Upon software initialization, the trainee sees a welcome screen as part of the “Game Start” action. Next, the “Demographics” action asks the trainee to input anonymized identification information into the system; this step enables the software and the research team to track the trainee utilizing the platform at a given time. In the Training Session, the “Training Instructions” action presents the trainee with brief written instructions of the tasks to be achieved. Subsequently, two concurrent actions occur in the PARS platform: “User Training” and “Training Data Serialization”. The “User Training” action proceeds with hazard-discovering tasks that the trainee is required to complete on each 360-degree image. As the trainee interacts with the platform, data is recorded by the “Training Data Serialization” action, at which time the recorded training data is encoded into JSON format to facilitate the transfer, storage, and retrieval from and to the database.

In the Assessment Session—represented by the “User Assessment” and “Assessment Serialization” actions—trainees first utilize an interface to identify hazards in each 360-degree image (“User Assessment”); simultaneously, the data inputs are serialized into JSON in the “Assessment Data Serialization” action. The recorded hazard data is then automatically graded during the “Data Verification” action using a set of defined answer keys stored within the platform. Then, the data is transferred for storage to the database in the “Assessment Data” action for later processing. The trainee traverses a series of 360-degree images until a final scene is reached, where the trainee is prompted to move into the Feedback Session. In the Feedback Session, the “User Feedback” action the assessment data is retrieved from the databased and the defined answer keys on the platform, comparing them to populate a feedback interface with the correct and incorrect answers. Once the trainee reaches this screen, the game is complete. In the “Game Completion” action, the user receives a message to restart the “Game Start” action for the next user or to end the game, exiting the application.

By utilizing the architecture described in this section and following the UML data management procedure, the Unity3D application can be used to learn, assess, and receive feedback regarding safety hazards hosted in the 360-degree augmented panorama of reality and the database. Although trainees experience a pre-assembled set of content that cannot be modified directly by them, potential future training creators can access the Unity3D application to add, manipulate, or replace the 360-degree images and layers of information augmented in each scene (e.g., text, objects, animations, etc.), developing their own customizable experience. This provides great flexibility for the proposed proof-of-concept, allowing content designers to potentially explore other educational and training alternatives beyond safety-related topics by simply changing the scope and materials loaded in the platform.

### 4.2. 360-Degree Panoramas: Capture, Visualization and Augmentation

**Figure 3:**
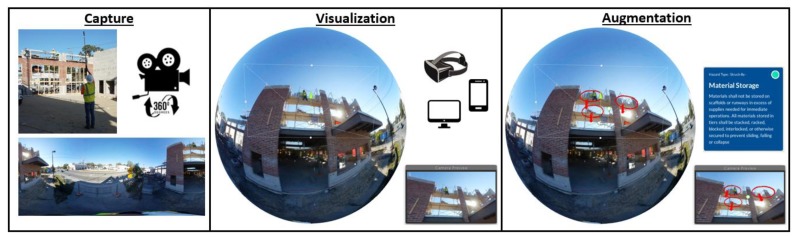
360-Degree Panoramas Capture, Visualization, and Augmentation.

Assembling 360-degree panoramas requires capturing images of the real environment to populate the virtual environment, as illustrated on Figure 3. 360-degree image capturing entails the creation of an equirectangular projection. To obtain this 360-degree capture as a 2D projection, a panoramic camera with multiple fish-eye lenses is used (e.g., Ricoh Theta V—Ricoh Company, Ltd, Tokyo, Japan; Insta360 One—Shenzhen Arashi Vision Co., Ltd, Shenzhen, China; Samsung Gear 360 —Samsung Group, Seoul, South Korea; etc.). Alternatively, multiple shots from a traditional camera (DSLR or Mirrorless) can be stitched to create an equivalent equirectangular image. In both approaches, the equirectangular projection requires the use of computer software to stich each individual image into a single picture; the software resolves the distortions introduced during the capturing process and maps the 360-degree spherical coordinates onto planar coordinates. Subsequently, the game engine—such as Unity3D^®^—remaps the equirectangular images into the spherical coordinates to render 360-degree image.

In the produced 3D virtual environment, trainees can explore the images to observe focus areas in detail. The augmentation process is performed by the training creator using the Unity3D game engine application software, in which data, objects, animations, or sounds can be superimposed into the 360-degree panoramas; augmenting the information displayed by importing these graphical or auditive assets into the scenes. In this study, the purpose of these augmentations is to communicate safety concepts using supplementary features that enhance users’ understanding of a written description from OSHA’s manuals. The resulting augmented 360-degree panoramic scenes can be transferred to different devices for visualization. This process provides trainees with access to the 360-degree panoramic imaging in a variety of devices such as PCs, laptops, handheld devices, and head-mounted displays (HMD). For this research, PCs were targeted as the primary device of analysis, as these are easily accessible and do not require any special setup. A mouse and keyboard setting were utilized to enable trainees to explore using drag-and-drop gestures in the 360-degree images interface and point-and-click gestures in the HIP interface. The 360-degree panoramas are also accessible using online cloud technologies, which enable real-time feed and big data analysis [46].

### 4.3. Hazard Recognition Sessions: Training, Assessment, and Feedback

**Figure 4:**
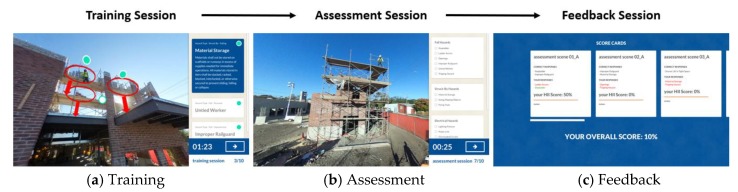
Hazard-Recognition Sessions: (**a**) Training, (**b**) Assessment, and (**c**) Feedback.

To effectively utilize the 360-degree panoramas and the safety augmentations as a hazard-recognition training platform, the content developed was structured into three distinct sessions: Training, Assessment, and Feedback (Figure 4). The Training Session (Figure 4a) focuses on leveraging the augmentations contained in the 360-degree panoramas to facilitate the retrieval and retention of safety-related information. Each image includes visual cues—such as dots, circles, or arrows—that alert the user to augmentations within the image. By allowing trainees to freely explore safety content in the panoramic spaces, the platform fosters active learning.

The Assessment Session (Figure 4b) concentrates on utilizing the 360-degree panoramas to evaluate the knowledge acquired by the trainees in the Training Session. In the Assessment Session, trainees are asked to identify hazards in a series of 360-degree images that do not present any type of augmentation. There, the hazard recognition is left entirely to the trainee by not providing the visual cues presented on the previous session. Once the trainee concludes the evaluation, instantaneous feedback appears in the Feedback Session (Figure 4c). In this final session, users evaluate their successful responses from the Assessment Session alongside any incorrect or missed hazards to cement learnt knowledge and to improve comprehension of safety hazards.

### 4.4. Hazard Recognition Evaluation: Hazard Identification Index and Grading

The evaluation of the trainees’ hazard-recognition skills is performed using the hazard identification index (*HII*) developed by Carter and Smith [20]. The *HII* (1) offers a method to score hazard identification quantitatively in the context of both the identification and the assessment of hazards. The *HII* is calculated for each trainee as the ratio:

(1) $$HII_{j}\;=\;\frac{H_{i}}{H_{total}}$$

where $H_{i}$ is the number of identified hazards, and $H_{total}$ is the total number of hazards present in each 360-degree image (*j*). The number of hazards identified by the trainee ($H_{i}$) will be impacted by the level of conceptual comprehension the trainee gained during the Training Session. To successfully reflect the understanding of the trainee, a grading system assigns a value to each potential response. Trainee hazard identification can correspond to three cases:

- *Correct identification or rejection (CIR):* Trainee identifies correctly a hazard as present or as not present in the image.
- *Incorrect identification (II):* Trainee identifies a hazard as present in the image, but the hazard it is not actually contained in the image. Incorrectly identified hazards are analogous to a false positive or Type I error.
- *Missed identification (MI):* Trainee identifies a hazard as not present in the image, but the hazard is in fact contained in the image. Missed hazards are analogous to a false negative or Type II error.

Calculating the number of identified hazards is accomplished by combining the concepts associated within the training to the three previously defined cases, assigning a positive point for each *CIR* and penalizing a proportion of the *II’s* and *MI’s* with negative points. As no literature was found regarding the appropriate percentage of penalization for *II’s* and *MI’s*, the research team assumed a value of 50 percent for each of these categories, thereby weighting both II and MI errors as equally detrimental to the assessment score. The proposed equation for the calculation of $H_{i}$ is defined as:

(2) $$H_{i}=\;\sum CIR-\left[\left(\sum II\cdot 0.5\right)+\left(\sum MI\cdot 0.5\right)\right]$$

To compute the overall hazard identification index ($\bar{HII}$) across the scenes for each trainee, the mean is calculated using each index previously computed ($HII_{j}$) divided by the total number of hazards present on the scene (N):

(3) $$\bar{HII}\;=\;\frac{\sum_{j=1}^{N}HII_{j}}{N}$$

### 4.5. Graphical User Interface

**Figure 5:**
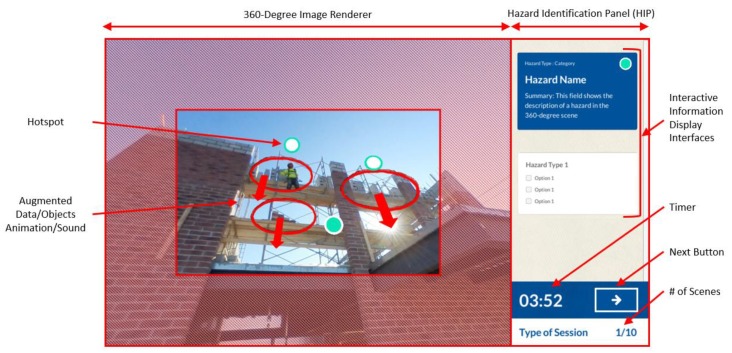
PARS Platform Application Graphical User Interface.

Trainees must constantly interact with the PARS platform on each of the sessions to learn, evaluate, and obtain feedback about the hazards present in the 360-degree images. These interactions are driven by the platform’s graphical user interface, which enables data input and output. Figure 5 illustrates the most important user interfaces the trainees encounter while performing the hazard-recognition tasks throughout the sessions. As discussed above, within the application, trainees have access to two different areas within the scene screen: the 360-degree image renderer and the hazard identification panel (HIP). The 360-degree image renderer allows the trainee to actively explore the scene by using drag-and-drop gestures with different pointing devices or finger movements. In this area, graphical representations of the hazardous conditions are displayed using augmentations (data, objects, animations, or sounds). A special type of object augmentation in the PARS platform is the Hotspot. These are safety data-rich locations annotated with graphics using different colors to direct the attention of the trainee to a hazardous situation. The content and position of these augmentations, including the marker, enhances the trainees’ contextual understanding about the safety-related topics (e.g., activity, objects, or persons) in the location.

**Figure 6:**
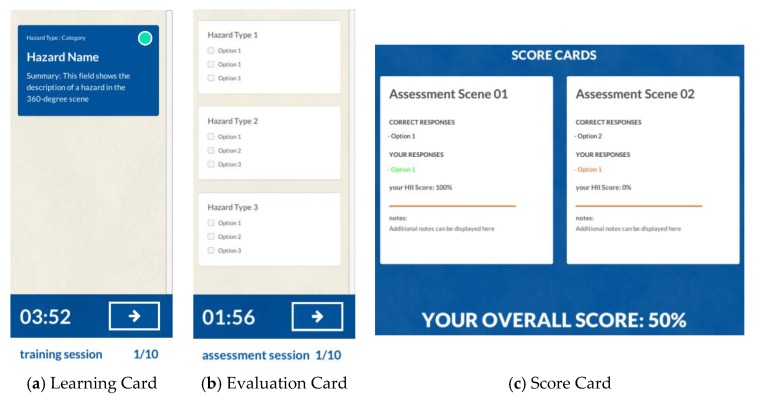
Hazard Identification Panel: (**a**) Learning, (**b**) Evaluation and (**c**) Score Cards.

The HIP facilitates trainee interaction with the descriptive information that accompanies the hazards displayed in the 360-degree image renderer. The HIP employs three different interfaces depending on the type of session (Training, Assessment, or Feedback). Figure 6 displays the HIP’s types of information, interaction, and layout for each of the different sessions. For the Training Session, the HIP utilizes the learning card (Figure 6a) to contain descriptive safety information. The learning cards directly link the graphical representation of a hazard to the descriptive information in a hotspot. When a trainee uses a point-and-click gesture on a hotspot or on the learning card, the game camera is automatically directed to the augmentation and shows the contained information. The learning card information has three layout levels: hazard category, hazard name, and hazard summary.

The hazard category indicates the type of hazard the card contains according to a hazard classification scheme (e.g., fall hazard, struck-by hazard, electrical hazard, etc.). The hazard name defines the specific source of the hazard by assigning a distinctive term that outlines the content scope (e.g., a “fall hazard” will include an untied worker, unprotected edges, holes, etc.). Finally, the hazard summary elaborates on the exact context presented in the 360-degree image and provides descriptive information for the trainee to fully understand the hazardous condition.

For the Assessment Session, the HIP uses the evaluation card (Figure 6b) to contain all the possible answers for the hazard-recognition tasks. The evaluation cards use a checkbox interface to collect the trainee responses for each scene. Each card layout contains the hazard category as its title and the hazard names covered within the category as options to be selected by the users. The user responses collected from these cards are linked to the score cards (Figure 6c) in the Feedback Session. In the Feedback Session, the HIP displays for each assessment scene: the correct answers, the user responses graded and color coded (green as correct, and red as incorrect), the hazard identification index, and additional notes. An overall hazard identification score is displayed below the score cards to deliver a notion of the user understanding across the different evaluated scenes. In general, the HIP also contains a timer that specifies the time used for the session, a next button to advance to the subsequent scene, an indicator of the type of session currently in use, and a counter that shows the current number of the scene.

## 5. Materials and Methods: Building a PARS Proof-of-Concept Using OSHA’s Focus Four Hazards

The Occupational Safety and Health Administration (OSHA) and OSHA’s Susan Harwood Grant (SHG) has produced extensive collections of regulation and training materials related to hazard identification specific to the construction industry. These initiatives have identified four leading causes of fatalities in the construction industry: Fall hazards, Struck-By hazards, Caught-in or -between hazards, and Electrical hazards. Due to the importance of these four categories, this study used the OSHA focus four as the basis for the proof-of-concept study’s hazard classification scheme. Safety augmentations hosted on the 360-degree images were specifically designed around the focus four categories, displaying descriptive information analogous to OSHA’s regulations and graphical representations of the hazardous conditions equivalent to SHG materials.

To capture the visual data necessary to conduct this study, ten active construction jobsites were visited, and more than 600 360-degree panoramas were obtained. The captured images aimed to graphically demonstrate hazardous scenarios in the real-world context of construction jobsites. To accurately determine the focus four hazards, present in each image, an advisory board of construction safety experts were consulted (1) to determine the best images to use in across the sessions and (2) to build the correct hazard answer keys for the assessment session. To be included in this panel, participants needed to be certified safety professionals (CSP) with more than ten years’ experience. Ten safety managers were recruited to serve on this panel and identified hazards in each image.

Out of the pool of previously captured scenes, the advisory board selected the twenty panoramic images that were most representative of the focus four hazard scenarios. Of these twenty images, ten were selected for the Training Session. Based on the answer keys provided by the advisory board, the research team augmented each image in the software to highlight the focus four hazards, as described next. The remaining ten images were used in the Assessment Session, where the answer keys determined by the advisory board were used as the baseline to compute the HII values for each trainee in the Feedback Session.

**Figure 7:**
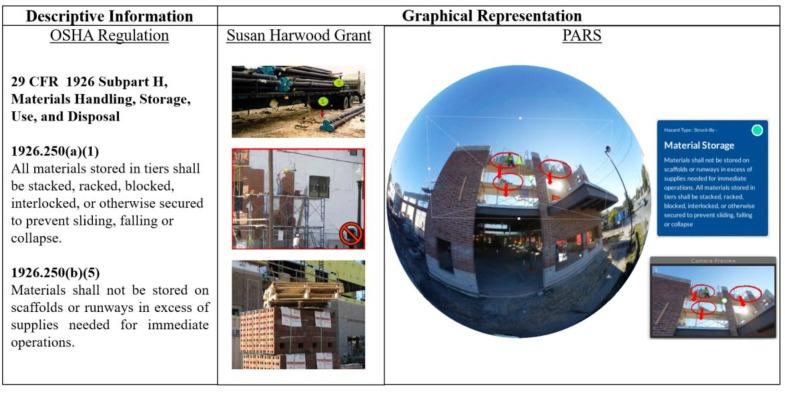
Safety Augmentation—Material Storage.

Figure 7 illustrates an example of the augmentations the research team undertook. To annotate the Struck-By hazards—specifically scaffold material storage—visible in the 360-degree image, the research team used the OSHA’s regulation—descriptive text—to define the material storage hazard. Concurrently, the team used SHG-type visualizations—hot-spots or graphical markers—to locate points of interest in the scene. In the PARS platform, these descriptive and graphical augmentations appear superimposed over the image to demonstrate the potential direction of a material stack collapse. By combining the graphical elements and the descriptive elements in the safety augmentations, the trainee can observe a unified perspective of a commonly disaggregated, complex hazardous situation, such as the scaffold material storage.

## 6. Usability Evaluation

To assess the features developed in the training platform, this study conducted a usability test with potential users of the technology. Usability studies are often conducted in Human-Computer Interaction research studies to discover what are the aspects most concerning in a new platform that has never been test with users such as PARS. Farrell [47] indicates that usability studies aid to reveal how user understand the features in a software, and highlights issues that make users unsatisfied with the system. These types of studies also show potential problems that need revisions in the platform, and displays which tasks take too long to accomplish for an average user.

### 6.1. Usability Methodology

**Figure 8:**
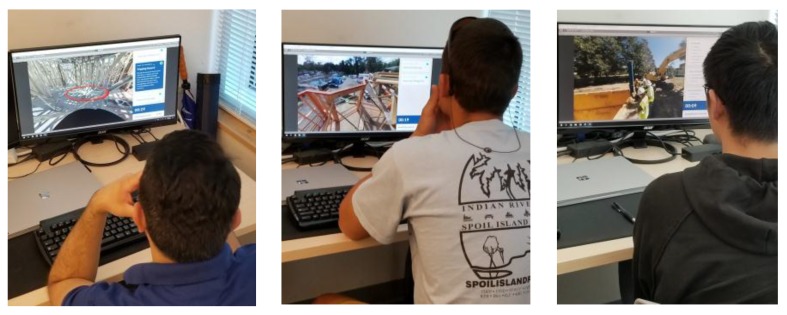
Subjects using the training platform.

To evaluate the usability of the platform, its function as a training and assessment tool, and user satisfaction, this study collected feedback and HII scores from real trainees (Figure 8). Data were collected from University of Florida’s (UF) students using three different instruments: a (1) pre-test survey; a (2) hazard identification test; and a (3) post-test survey. The data-collection process took 20 to 25 min in total and was performed in a quiet, air-conditioned room, where the participants had no interruption during the Training, Assessment, and Feedback Sessions. In the (1) pre-test survey, the research team collected demographic information regarding the trainees, including age, gender, educational level and background, and previous experience/knowledge in construction. Also, participants used a four-point Likert scale to self-assess their level of understanding about the following topics: virtual/augmented reality and 360-degree panoramic imaging.

In the (2) hazard identification test, the trainees were asked to use the training platform with the objective of assessing their knowledge of the different hazards present in the images. Accordingly, the subjects engaged the PARS platform in keeping with the three different sessions defined on Section 4.3, above. Each session was successive and included within the developed platform. In the Training Session, ten panoramic images were shown to the trainees, each containing visual cues signaling augmentations available to engage. The exposure to each image was limited to 1 min, and the number of hazards appearing on the image varied from 1 to 4 hazards. In this session, the trainees had to actively traverse, discover, and interact with safety information in the panoramic site scenes to learn the focus four hazard content presented. Subsequently, the Assessment Session showed ten consecutive panoramic images without augmentations; the subjects were only given 30 s explore and identify the hazards. In this session, trainees were requested to identify all the hazards within the scope of the focus four hazards previously described in the Training Session. The data were collected within this session by automated processes in the platform. Once the trainees complete the hazard identification in the assessment session, instantaneous feedback was presented to them in the Feedback Session. For each image, the correct answers were displayed alongside the subject’s answers and the subject’s HII, as calculated using the formula proposed in Section 4.4, above.

In the (3) post-test survey, trainees provided feedback regarding the usability of the platform. These data were collected with the goal of improving the platform for later testing on construction workers and professionals. The ease of use and user satisfaction about the platform was evaluated by using a modified version of the Questionnaire for User Interface Satisfaction (QUIS), developed by the University of Maryland [48]. QUIS (version 7.0) is a validated survey designed to accurately test ease-of-use and satisfaction for computer software. In this study, the adapted QUIS survey sections included: overall user reactions, responses regarding in-screen elements (e.g., quality of image, quality of written characters, amount of time, and text provided), safety terminology and system information, ease of learning to operate the platform, and hazard-identification content information. Each post-test survey section used a nine-point Likert scale, with the endpoints scores representing opposite subjective qualificative adjectives (e.g., Terrible (Score: 1)–Wonderful (Score: 9)). This feedback system using Liker-scale questions allows the trainees to express their thoughts and opinions regarding the platform [48]. Moreover, at the end of each section, the survey provided an area for open-ended comments to allow trainees to provide supplementary explanations for their scores.

### 6.2. Participants: Demographics, Industry Experience, and Technology Knowledge

The sample size obtained for this usability study was 30 UF students. The resulting demographics are shown in Table 3. As it can be observed, most participants were male (90%), were less than 30 years old (70%), had more than one year of experience in the construction industry (63%), had an OSHA-30 certificate (87%), and had experience with 360-degree panoramas (80%).

## 7. Results and Discussion

### 7.1. Platform User Satisfaction and Ease of Use

The perspectives from the trainees regarding the usability of the platform and how satisfied they were with the overall experience was captured using an adapted version of the QUIS survey, as described in the previous section. Here, we address each part of this survey individually:

#### 7.1.1. Part 1—Overall User Reactions

The trainees’ reaction to the platform was positive, with response values aggregating at the positive end of the scale (Table 4). A few participants expressed some frustration with the Assessment Session due to the limited amount of time they received to identify hazards in the scene; for example, one noted that “*more time should be added to identify problems*”. On the other hand, several of the trainees provided insights into the most important attributes of the platform. For example, a trainee noted that “*This was a great method to view an entire jobsite*,” and another mentioned that “*(the platform is) good and helpful to visualize hazards*”.

#### 7.1.2. Part 2—Screen

Trainee feedback presented a positive trend in response to the visual elements of the platform (Table 5). In particular, trainees reviewed the written text on the screen, the image quality, the amount of information displayed, the sequence, and the progression of the platform using positive adjectives in the scale. For example, a trainee supported his scores by commenting that the elements on the platform were “*pretty clear and neatly displayed*”. Nevertheless, some other trainees indicated that the “*images could be clearer*” and that “*sometimes the picture would become fuzzy for several seconds while rotating*”.

#### 7.1.3. Part 3—Safety Terminology and Platform Information

High scores were obtained in this section of the survey. The trainees expressed that the platform presented well-defined safety terminology and that the information was clearly displayed on the platform (Table 6). Supporting comments were provided by the trainees; for example, one participant noted that “*terminology is consistent with OSHA*”.

#### 7.1.4. Part 4—Learning

The trainees’ responses in relation to the ease of learning how to use the platform, the steps to complete the tasks, and the feedback elements within the platform were all positive. As shown on Table 7, the scores obtained were towards the high end of the scale. Although these positive scores were obtained, one trainee indicated that the platform could be improved by “*showing the results on feedback as pictures so the user can understand his/her error better*”, and another recommended that the “*score cards could be categorized so that the user can understand deficient areas better*”.

#### 7.1.5. Part 5—Hazard Identification Using the Platform

The hazard-identification tasks in the platform received mixed positive and negative scores from the trainees (Table 8). The trainees perceived the platform as a helpful method to perform safety training and rated the Training Session with positive scores. Animations presented in the images were scored especially high, as these enable the trainees to easily visualize the hazards. Nevertheless, the Assessment Session received low scores due to the users’ difficulties in identifying hazards and due to the limited amount of time users had to recognized hazards. Trainees’ comments indicated that “*time for training and assessment were either too short or too long*”, and that “*more time should be given (for the whole platform)*”.

### 7.2. Hazard Identification Index

Each participant’s HII was calculated across all the 360-degree images. This index reflects the ability of each trainee to identify hazards within the context of the developed platform. The index was computed using the formulation defined in Section 4.4. As this is the first study conducted using the developed platform and the proposed *HII* index, the *HII* calculation was obtained assuming that 50 percent penalties from incorrect identification of hazard (*II*) or missed identification of hazards (*MI*); that is a deduction of points for half the value for each II or *MI* answer provided from the trainee in the CIR of a particular scene. The outcomes of the HII index calculation of this research provides an initial baseline scoring for future calibration of the proposed approach. As a result, participants recognized an average 30% of the hazards displayed throughout the entirety of the Assessment Session. These results are consistent with previous studies that indicated that several hazards are constantly unidentified on construction sites [20,21]. However, this low level of identification success is distressing, since unidentified hazards are a major source of incidents on construction jobsites.

Since trainees had diverse backgrounds and industry experience, the HII was also computed for each individual image to provide additional insights into these results. Table 9 presents the average HII for each image, the standard deviation (STD), the hazards present on the panoramic image, and the type of focus four hazard. Overall, each image averaged an HII less than 50%, with very large standard deviations. The focus four category that had the widest ranging score was Struck-by hazards (High: 47.5–Low: 5.0), followed by Electrical hazards (41.6–18.9), Fall hazards (41.6–18.9), and Caught-in/-between hazards (47.5–21.7). The wide HII range for Struck-by hazards suggests that these hazards can be challenging to spot in a real construction jobsite. Inversely, Caught-in/-between hazards have a lower spread, indicating that these might be simpler to identify in a construction site. Nevertheless, these results are only indicative of the environments captured in the 360-degree images and will greatly vary depending on the specific context and location.

## 8. Research Limitations

This proof-of-concept study was of an exploratory nature, offering a general overview of the variables and factors affecting the topic and of the experiences reported by the participants. This research has limitations due to the sample size collected and the target population for the study. Due to the number of participants, the results presented in this document cannot be used to provide statistical generalizations. Moreover, these results only provide insights for the population sampled—students that majored construction management; indicating that other populations such as construction workers and professional might reveal different results. Nevertheless, for usability purposes research has found that 30 users can identify up to 99% of the problems in a software [49]. Additionally, the scope of the hazards selected for this study only covers a few select cases of the focus four due to the limited number of pictures. Accordingly, the approach presented in this research is not universal but rather meant to provide insights into how to content creator can design hazard-identification materials in future studies that use the 360-degree panorama. Moreover, the new approach for scoring the HII lacks any form of validation since it is the first in its kind, but it provides a baseline score for future researchers using this method. Finally, the device selected for this proof-of-concept study was a PC, which does not provide the highest level of immersion possible for participants. The use of head-mounted displays might modify the results found on this research as well as change the design considerations made for the hazard-identification material in this study.

360-degree panoramas as a medium have several limitations regarding image quality, static vantage point, and stitching parallax. As the technology to capture 360-degree panoramas is very recent, the image quality of commercially available cameras is often not comparable of that of traditional photography or videography, and therefore this technology produces resolutions inferior to those of more traditional equipment [46]. Furthermore, due to the nature of being a photographic technology, the panoramas have a static vantage point. This constraint limits the exploration of the data to visual rotation, allowing for only prearranged visual translation using videos. Moreover, while the algorithms employed in panoramic stitching techniques and methods are robust, parallax issues are still present on the images. These issues are often visible for object that are very close to the focal point of the camera or sometimes at the intersection of the stitching lines. The parallax issues are impossible to remove entirely, but they can be managed to the point of being largely imperceptible.

## 9. Conclusions 

The use of 360-degree panoramas to create a true-to-reality view of the actual construction site can provide an interactive, true-to-reality safety training experience for construction workers and professionals. This study described the development a platform for augmented 360-degree panoramas of reality and defined a user-experience that is conducive for teaching hazard-identification skills based on OSHA and SHG training materials. To validate the usability and interface of the platform, a usability test was conducted. The finding of this research showed that hazards were identified by the training participants in an average of 30%. Constructive feedback was obtained concerning the usability of the platform. The study participants stated in general that the platform was easy to use, easy to learn how to operate, and noted that on screen augmentations aided them to locate the hazards in the panoramic scenes. However, the participants expressed that numerous enhancements need to be attended in the platform to improve the experience, specifically indicating that the time limits posed on the assessment session were too short.

Future research should investigate using augmented 360-degree panoramas of reality to provide trainees with hazard-identification knowledge about other types of hazards or using a different scheme for categorizing hazards. Further, this training methods should be compared to a more traditional intervention and to VR training methods to understand how effective the PARS platform is in transferring safety-related knowledge. Additionally, a larger sample size would be required to perform this study with a detailed statistical analysis of participants’ hazard-identification skills, and researchers may benefit from testing this platform’s usability on other devices (e.g., tables or HMDs) to find the most conductive approach to delivering the safety information. Finally, the platform should be tested with non-English speakers to assess how 360-degree immersive environments might have an impact on the hazard-identification for trainees of different ethnicities or nationalities. The newly proposed approach for evaluating HII requires additional investigation for validation and to investigate the effect on the decision-making process of trainees while identifying hazards. Finally, the incorporation of 360-degree videos and spatial audio in the platform should be explored to determine their impact on trainees’ hazard identification skills.

## Figures and Tables

**Table 1:** VR Applications for Safety Training.

Authors	Purpose
Li et al. [19]	Used a game engine combined with a Wii game controller to produce a multiuser VR training program. Users were able to practice safe crane dismantling procedures.
Guo et al. [24]	Designed a collaborative, multi-user game that allowed trainees to navigate and perform construction operations for safety education in tower crane, mobile crane, and pile driver activities.
Dickinson et al. [25]	Conducted a robust experiment engaging trade students in a serious game on trench safety, which included fall, struck-by, and caught-in hazards.
Lin et al. [26]	Developed a serious game aimed at being immersive, interactive, and entertaining to test on users’ hazard identification skills
Le et al. [27]	Created a collaborative platform that replicated real-world accidents. Students learned common industry safety practices while roleplaying and social interacting in the digital environment.
Pedro et al. [28]	Devised a serious game for university students to learn of safety material, rules, regulations, and hazards through the interaction using VR and smart devices.
Bosché et al. [29]	Employed a head-mounted display simulation in conjunction to real-world prompts to simulate jobsite conditions. Scaffolds or beams situations were replicated to train students how to react to hazards.

**Table 2:** 360-Degree Panorama Applications for Safety Training.

Authors	Purpose
Jeelani et al. [41]	Used of 360-degree panoramic images for simulating personalized accidents to train construction workers. The pilot system showed that such 360-degree panoramic images provided high degree of presence.
Eiris et al. [42] and Eiris et al. [43]	Developed a virtual safety training environment using augmented panoramas of reality. The platform enabled trainees to traverse a construction site, obtaining comprehensive information about the environment. The was platform tested with real trainees, discovering that fall hazards were recognized in 52% (average) of images by the study participants.
Pham et al. [44] and Pham et al. [45]	Created a learning system for improving the safety education. In the learning system, a virtual site visit was conducted to identify hazardous situations in construction jobsite employing 360-degree panoramas. The preliminary results of this system found no statistical differences of the scores of students that used the 360-degree panoramas to learn about safety hazard in comparison to students that visited the real construction jobsite to perform the same tasks.

**Table 3:** Demographics of participants.

Variables	Categories	Frequency (Percentage)
Age	19–25	18 (60%)
	26–30	9 (30%)
	31–40	3 (10%)
Gender	Male	21 (70%)
	Female	9 (30%)
Educational background	Const. Mgmt.	19 (63%)
	Engineering	9 (30%)
	Architecture	2 (6%)
Academic rank	Junior	10 (33%)
	Senior	3 (10%)
	Master’s	6 (20%)
	PhD	11 (37%)
Experience in construction	Less than 1 year	11 (37%)
	1 to 2 years	10 (33%)
	2 to 4 years	3 (10%)
	4 to 10 years	6 (20%)
Safety-related work experience	Yes	4 (13%)
	No	26 (87%)
University-level safety coursework	Yes	26 (87%)
	No	4 (13%)
OSHA certification	No Certificate	3 (10%)
	OSHA-10	1 (3%)
	OSHA-30	26 (87%)
Experience with VR/AR	None	5 (17%)
	Some	11 (37%)
	Fair	14 (47%)
	Competent	0 (0%)
Experience with 360-degree panoramas	None	6 (20%)
	Some	10 (33%)
	Fair	14 (47%)
	Competent	0 (0%)
Understating general concepts of construction safety management	None	0 (0%)
	Some	8 (27%)
	Fair	18 (60%)
	Competent	4 (13%)
Understanding of OSHA regulations	None	1 (3%)
	Some	6 (20%)
	Fair	18 (60%)
	Competent	5 (17%)

**Table 4:** Results of QUIS Part 1—Overall User Reactions.

Question	Scale: 1–9	Mean (STD)
Q1. Overall reactions to the system:	Terrible/Wonderful	7.0 (1.2)
	Frustrating/Satisfying	6.2 (1.7)
	Dull/Stimulating	6.9 (1.9)
	Difficult/Easy	6.0 (2.0)
	Rigid/Flexible	6.1 (1.7)

**Table 5:** Results of QUIS Part 2—Screen.

Question	Scale: 1–9	Mean (STD)
Q2. Quality of the 360-degree image:	Fuzzy/Sharp	6.9 (1.2)
Q3. Characters on the computer screen:	Hard to Read/Easy to Read	8.0 (0.8)
Q4. The written character in the screen are:	Fuzzy/Sharp	7.8 (1.1)
Q5. Character shapes (fonts):	Barely Legible/Very Legible	8.2 (1.0)
Q6. Highlighting on the screen:	Unhelpful/Helpful	8.2 (1.0)
Q7. Amount of information displayed on the screen:	Inadequate/Adequate	7.7 (1.1)
Q8. Arrangement of information on the screen:	Illogical/Logical	7.3 (1.5)
Q9. Sequence of screens:	Confusing/Clear	7.5 (1.5)
Q10. Progression of tasks:	Confusing/Clear	7.3 (1.4)

**Table 6:** Results of QUIS Part 3—Safety Terminology and Platform Information.

Question	Scale: 1–9	Mean (STD)
Q11. Use of safety terminology through the platform:	Inconsistent/Consistent	7.9 (1.4)
Q12. Safety terminology relates well to the work you are doing in the platform:	Never/Always	7.3 (1.3)
Q13. Safety terminology on the screen:	Ambiguous/Precise	7.2 (1.9)
Q14. Messages which appear on screen:	Inconsistent/Consistent	7.9 (1.8)
Q15. Position of instructions on the screen:	Inconsistent/Consistent	7.8 (1.4)
Q16. Platform keeps you informed about what you are doing:	Never/Always	7.3 (1.3)

**Table 7:** Results of QUIS Part 4—Learning.

Question	Scale: 1–9	Mean (STD)
Q17. Learning to operate the platform:	Difficult/Easy	8.3 (0.9)
Q18. Tasks can be performed in a straight-forward manner:	Never/Always	8.2 (0.9)
Q19. Number of steps to complete all the tasks in the platform:	Too Many/Just Right	7.9 (1.3)
Q20. Steps to complete all the task in the platform follow a logical sequence:	Never/Always	7.4 (1.5)
Q21. Feedback on the completion step is:	Unclear/Clear	7.6 (1.3)

**Table 8:** Results of QUIS Part 5—Hazard Identification Using the Platform.

Question	Scale: 1–9	Mean (STD)
Q22. The use of the platform as a safety training method is:	Unhelpful/Helpful	7.6 (1.5)
Q23. Number of hazards present on each image in the *Training Session*:	Too Many/Just Right	6.8 (1.9)
Q24. The hazard content in the *Training Session* is:	Unclear/Clear	7.1 (1.7)
Q25. Usefulness of the animations present in the *Training Session:*	Unhelpful/Helpful	8.2 (1.1)
Q26 Amount of time to review the information in the *Training Session*:	Inadequate/Adequate	7.0 (2.0)
Q27. Identifying safety hazards in the *Assessment Session* is:	Difficult/Easy	5.2 (2.1)
Q28. Amount of time to identify hazards in the *Assessment Session*:	Inadequate/Adequate	4.1 (2.1)
Q29. The hazard options in the *Assessment Session* is:	Unclear/Clear	6.2 (2.1)

**Table 9:** Focus Four Hazard by HII and Image Number.

Image Number	Average *HII* (%)	Hazards in Image	Focus Four Hazard Type
9	47.5 (STD = 43.2)	“Cave In”, “Swing/Slipping Objects”	Caught-in/-between, Struck-by
8	41.6 (STD = 41.7)	“Lighting Fixtures”, “Tripping Hazard”, “Scissor Lift in Tight Space”	Electrical, Fall, Caught-in/-between,
6	40.0 (STD = 38.1)	“Floor Openings”	Fall
4	32.5 (STD = 41.1)	“Improper Guardrail”, “Swing/Slipping Objects”	Fall, Struck-by
10	32.2 (STD = 34.7)	“Cave In”, “Swing/Slipping Objects”, “Ladder Access”	Caught-in/-between, Struck-by, Fall
1	28.3 (STD = 37.0)	“Stepladder”, “Improper Guardrail”	Fall
2	28.3 (STD = 37.0)	“Improper Guardrail”, “Material Storage”	Fall, Struck-by
3	21.7 (STD = 38.7)	“Scissor Lift in Tight Space”	Caught-in/-between
7	18.9 (STD = 25.4)	“Untied Worker,” “Material Storage,” “Power Line”	Fall, Struck-by, Electrical
5	5.0 (STD = 15.3)	“Material Storage”	Struck-by
